# Weeding volatiles reduce leaf and seed damage to field-grown soybeans and increase seed isoflavones

**DOI:** 10.1038/srep41508

**Published:** 2017-01-30

**Authors:** Kaori Shiojiri, Rika Ozawa, Ken-Ichi Yamashita, Masayoshi Uefune, Kenji Matsui, Chigen Tsukamoto, Susumu Tokumaru, Junji Takabayashi

**Affiliations:** 1 Center for Ecological Research, Kyoto University, 2-509-3 Hirano, Otsu, Shiga 520-2113, Japan; 2 The Hakubi Center for Advanced Research, Kyoto University, Yoshida, Sakyo-ku, Kyoto 606-8501, Japan; 3 Hyogo Prefectural Technology Center for Agriculture, Forestry and Fisheries, Agricultural Technology Institute, Kasai 679-0198, Japan; 4 Department Agrobiological Resources, Faculty of Agriculture, Meijo University, Nagoya, Aichi 468-8502, Japan; 5 Graduate School of Sciences and Technology for Innovation (Faculty of Agriculture), Yamaguchi University, 1677-1 Yoshida, Yamaguchi 753-8515, Japan; 6 Graduate School of Agriculture, Iwate University, Ueda, Morioka 020-8550, Japan; 7 Kyoto Prefectural Agriculture, Forestry and Fisheries Technology Center, Kameoka, Kyoto 621-0806, Japan

## Abstract

Field experiments were conducted over 3 years (2012, 2013, and 2015), in which half of the young stage soybean plants were exposed to volatiles from cut goldenrods three times over 2–3 weeks, while the other half remained unexposed. There was a significant reduction in the level of the total leaf damage on exposed soybean plants compared with unexposed ones. In 2015, the proportion of damage to plants by *Spodoptera litura* larvae, a dominant herbivore, was significantly less in the exposed field plots than in the unexposed plots. Under laboratory conditions, cut goldenrod volatiles induced the direct defenses of soybean plants against *S. litura* larvae and at least three major compounds, α-pinene, β-myrcene, and limonene, of cut goldenrod volatiles were involved in the induction. The number of undamaged seeds from the exposed plants was significantly higher than that from unexposed ones. Concentrations of isoflavones in the seeds were significantly higher in seeds from the exposed plants than in those from the unexposed plants. Future research evaluating the utility of weeding volatiles, as a form of plant–plant communications, in pest management programs is necessary.

In response to herbivory, plants emit a specific blend of volatiles that may attract carnivorous natural enemies of the herbivore^1^,^2^. Furthermore, volatiles from herbivore-damaged plants may induce direct and/or indirect defenses against herbivores in neighboring undamaged plants (volatile receivers)^3^,^4^,^5^. Such a phenomenon is called “plant–plant communication”. For example, several defensive genes were expressed in undamaged lima bean leaves only after exposure to volatiles from conspecific plants that had been damaged by two-spotted spider mites (*Tetranychus urticae*), and the subsequent mite damage was significantly less on these plants than on plants previously exposed to volatiles from undamaged plants^6^. In addition, undamaged lima bean plants that had been exposed to volatiles from *T. urticae*-damaged plants attracted more predatory mites *Phytoseiulus persimilis* than those exposed to undamaged plant volatiles^7^,^8^.

Plant–plant communication is also mediated by volatiles from artificially damaged plants^3^,^4^,^5^. Sagebrush plants (*Artemisia tridentate*) exposed to volatiles from artificially damaged conspecifics suffered less herbivory than those exposed to volatiles from undamaged ones^9^. Similarly, young undamaged seedlings of *Chrysanthemum cinerariaefolium* have increased amounts of pyrethrins when exposed to volatiles from artificially damaged conspecifics^10^. *Cotesia glomerata*, a parasitoid of cabbage white butterfly larvae (*Pieris rapae*), was more attracted to *Arabidopsis thaliana* plants that had been exposed to volatiles from artificially damaged conspecifics plants than to those exposed to undamaged plants^11^,^12^. Plant–plant communication mediated by volatiles has also been reported between artificially damaged heterospecific plants^13^,^14^,^15^.

Weeding is frequently performed to remove plant species that harm or compete with crops. Weeding results in the emission of large amounts of volatiles from cut weeds^16^, and thus may cause plant–plant communication, which could influence the defenses of neighboring commercial crops. However, this possibility has not been tested. To test this, we conducted 3-year field experiments using soybean plants (*Glycine max*) as crops and goldenrod (*Solidago altissima*) as the surrounding weed species.

## Results

### Leaf damages

**Figure 1:**
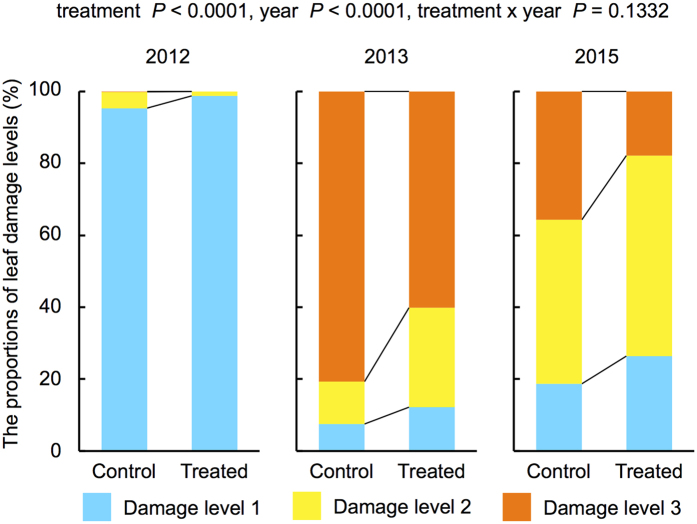
Proportions of damaged leaves in 3 years [Level l (0–10%), Level 2 (10–25%) and Level 3 (>25%)]. Treated: soybean plants exposed to volatiles from cut golden rods. Control: unexposed. GLMMs were applied to estimate the effects of the treatment, year, and their interaction on the proportions of damaged leaves.

We exposed soybean plants to volatiles from cut goldenrods for 2–3 weeks at their early growing period (20–30 cm high; around July) (Table S1), which was considered the treatment. In September, we randomly chose 18 (in 2012), 15 (in 2013) and 10 (in 2015) soybean plants from each plot (two treated and two control) in the experimental field (Figure S1) to determine the level of the leaf damage (Levels 1–3: see Methods section). In three years, common cutworms (CCW; *Spodoptera litura* larvae), *Medythia nigrobilineata* adults, Plusiinae larvae and Scarabaeidae adults were the dominant herbivores. We analyzed 3 years of data using a generalized liner mixed model (GLMM) and found that the treatment (exposure *vs.* control) and years were highly significant (treatment: df = 1; χ^2^ = 22.18, *P* < 0.0001; year: df = 2; χ^2^ = 824.73, *P* < 0.0001), while the interaction (treatment × year) was not significantly different (df = 2, χ^2^ = 4.03, *P* = 0.1332). These results indicated that over 3 years, the level of damage was significantly higher in the control soybean plants than in the exposed soybean plants (Fig. 1). The proportion of plants with whitened leaves damaged by CCWs was significantly lower (df = 1, χ^2^ = 3.96, *P* = 0.0465, GLM) in exposed plants (49.24%) than in unexposed plants (37.04%) in 2015.

### Effects of cut goldenrod volatiles on CCW performance

To test whether the volatiles from the cut goldenrods affected the performance of folivores through changes in the defense responses of the exposed plants, we conducted laboratory experiments measuring the growth rates of CCWs on an artificial diet and potted soybean plants. The CCWs’ weight gain when reared on an artificial diet for 7 d in an atmosphere containing cut goldenrod volatiles (treated CCWs: 660 ± 120 mg) did not differ significantly (df = 1,160, *F* = 0.067, *P* = 0.80; one-way randomized-block analysis of variance (ANOVA)) from those grown in a pure air atmosphere (untreated CCWs: 640 ± 48 mg). When CCWs were reared on soybean plants that had previously been exposed to the cut goldenrod volatiles for 4 days (treated plant-fed CCWs: 590 ± 120 mg), their weight gain was significantly less than those reared on unexposed plants (untreated plant-fed CCWs: 750 ± 130 mg) for 7 days (df = 1,73, *F* = 9.28, *P* = 0.0033; one-way randomized-block ANOVA).

### Effects of synthetic volatiles on CCW performance

**Figure 2:**
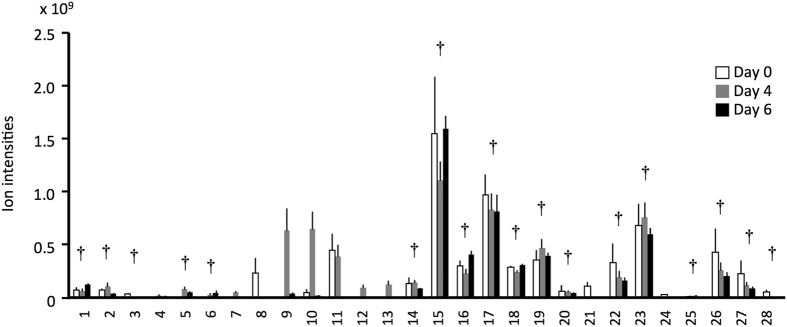
Volatiles emitted by artificially damaged goldenrod plants (N = 4). 1. pentan-2-one; 2. pentan-3-one; 3. unknown (1); 4. 3-methylbutan-1-ol; 5. 2-methylbutan-1-ol; 6. dimethyl disulfide; 7. (*Z*)-2-penten-1-ol; 8. unknown (2); 9. hexanal; 10. (*E*)-2-hexenal; 11. (*Z*)-3-hexen-1-ol; 12. (*E*)-2-hexen-1-ol; 13. hexan-1-ol; 14. thujene; 15. α-pinene; 16. camphene; 17. sabinene; 18. β-pinene; 19. β-myrcene; 20. phellandrene; 21. (*Z*)-3-hexen-1-yl acetate; 22. α-terpinene; 23. limonene; 24. *cis*-ocimene; 25. (*E*)-β-ocimene; 26. γ-terpinene; 27. α-terpinolene; 28. unknown (3). Daggers indicate that the amounts (ion intensities) were not significantly different among the three sampling dates (Welch’s one-way ANOVA).

To further clarify the active chemicals involved in the CCWs’ reduced performance, we conducted headspace analyses of the cut goldenrod volatiles. There were 28 compounds tentatively identified in the emissions of cut goldenrod (Fig. 2). The amounts of the 18 compounds did not change at the three sampling dates (Welch’s one-way ANOVA, *P* > 0.05). For a bioassay, we chose the major compounds α-pinene, β-myrcene, and limonene because they were commercially available. After 7 d of rearing, the weight gain of CCWs reared on soybean plants previously exposed to a blend of α-pinene, β-myrcene, and limonene for 4 d, at concentrations emitted from cut goldenrods was significantly less than those reared on unexposed control plants (treated plant-fed CCWs: 721 ± 413 mg, untreated plant-fed CCWs: 813 ± 414 mg; df = 1,118, *F* = 3.99, *P* = 0.048; one-way randomized-block ANOVA).

### Seed damage

**Figure 3:**
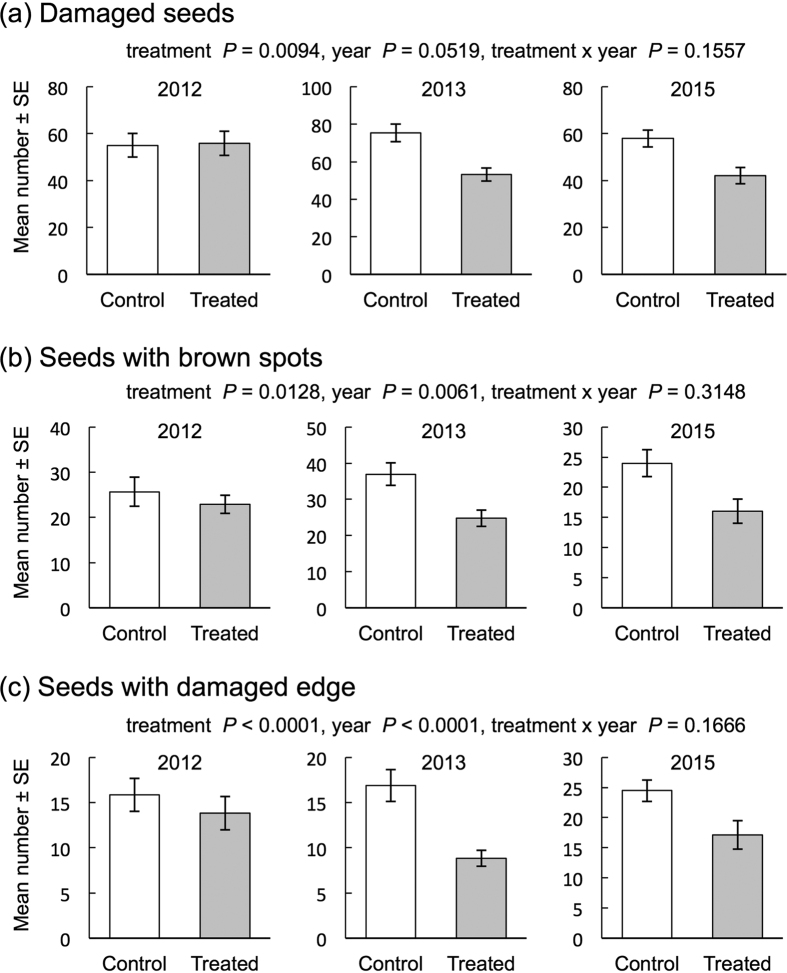
Number of damaged seeds (**a**), seeds with brown spots (**b**) and seeds with damaged edges (**c**). GLMMs were applied to estimate the effects of the treatment on the number of seeds with brown spots and those with damaged edges in each year.

Near November, we randomly chose 15 soybean plants from each field plot (two treated and two control) in the experimental field (Figure S1) to determine the number of damaged seeds and the total number of seeds. We first checked whether the number of seeds harvested was affected by the exposure. Over 3 years, the total number of seeds from the exposed soybean plants was not significantly different from that of the control soybean plants (treatment: df = 1; χ^2^ = 0.07, *P* = 0.7976; year: df = 2; χ^2^ = 2.79, *P* = 0.2479; treatment × year; df = 2, χ^2^ = 0.06, *P* = 0.9688; GLMM) (Figure S2). We then analyzed the number of seeds with damage (either brown spots, damaged edges or other symptoms). We analyzed 3 years of data using a GLMM and found that the treatment (exposure *vs* control; df = 1, χ^2^ = 6.74, *P* = 0.0094) and year (df = 2, χ^2^ = 5.92, *P* = 0.0519) were significant, while their interaction (treatment × year; df = 2, χ^2^ = 3.72, *P* = 0.1557) was not significantly different (Fig. 3a). Apparently, the number of damaged seeds in the treated soybean plants and that in the control soybean plants in 2012 were almost the same. The higher number of damaged seeds in 2013 and that in 2015 contributed to the results of GLMMs. Stinkbugs and lepidopteran larvae were the major seed predators during the 3 years. Damage caused by stinkbugs resulted in characteristic brown spots on the surface of soybean seeds. Thus, seeds having brown spots were judged to have been damaged by stinkbugs. Likewise, seeds with damaged edges were judged to have been chewed by lepidopteran larvae. We analyzed 3 years of data of the number of seeds with brown spots and damaged edge using a GLMM. We found that the treatment (brown spots: exposure *vs* control, df = 1, χ^2^ = 6.19, *P* = 0.01284; damaged edges: exposure *vs* control, df = 1, χ^2^ = 18.63, *P* < 0.0001) and year (brown spots: df = 2, χ^2^ = 10.20, *P* = 0.0061; damaged edges: df = 2, χ^2^ = 23.28, *P* < 0.0001) were significant, while their interaction (brown spots: treatment × year; df = 2, χ^2^ = 2.31, *P* = 0.3148; damaged edges: treatment × year; df = 2, χ^2^ = 3.58, *P* = 0.1666) was not significantly different (Fig. 3b,c). Over 3 years, the number of seeds with brown spots and damaged edge from the exposed soybean plants was lower than that of the control soybean plants.

### Seed isoflavones

**Figure 4:**
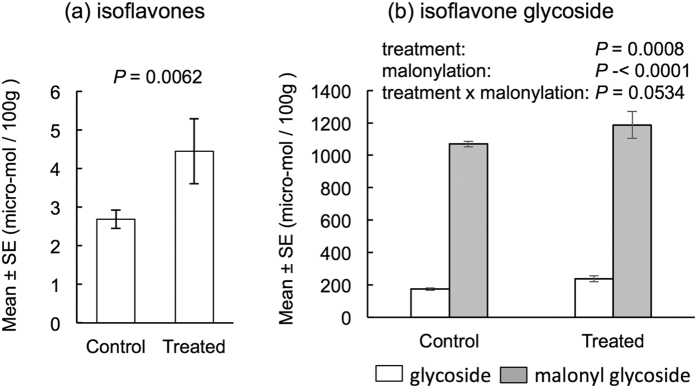
Amounts of isoflavones (**a**) and isoflavone glycosides and isoflavone malonyl glycosides (**b**). GLMs were used to estimate the effects of the treatment on the amount of isoflavones and to estimate the effects of treatment, malonylation, and their interaction on the amount of isoflavone glycoside.

Because the proportion of undamaged seeds increased in the exposed plants, we hypothesized that some of the biosynthetic pathways involved in the production of defensive secondary compounds in seeds were activated by the exposure. We focused on the production of isoflavones, characteristic secondary compounds in seeds, as an index of the activation of the phenylpropanoid pathway^17^. We used seeds of soybean plants in field experiments in 2013. The amounts of isoflavones (daidzein + glycitein + genistein) were significantly higher in seeds from exposed plants than those from unexposed plants [df = 1, χ^2^ = 7.49, *P* = 0.0062; generalized linear model (GLM)] (Fig. 4). GLM analyses of the amounts of glycosylated isoflavones and malonyl glycosylated isoflavones showed that treatment (df = 1, χ^2^ = 11.26, P = 0.0008) and malonylation (glycoside or malonyl glycoside) (df = 1, χ^2^ = 81.15, *P* < 0.0001) were both significantly different and that the interaction (treatment × malonylation: df = 1, χ^2^ = 3.73, *P* = 0.0534) was marginally significant.

## Discussion

We conducted field experiments over 3 years (2012, 2013, and 2015) and showed that exposing young soybean plants to cut goldenrod volatiles in the early growing period (July) only three times over 2–3 weeks significantly reduced the damage caused by folivores until September and by seed predators (except for 2012) until nearly November. Thus, we concluded that volatiles from cut goldenrods positively affected the defense levels of early stage soybean leaves and seeds. The effects of the exposure lasted until the seed-production stage. This was the first case study in which plant–plant communication resulted in a decrease in the damage level to leaves and seeds.

Undamaged plants that have been exposed to volatiles from artificially damaged plants have better defenses against folivores^4^,^5^. Under laboratory conditions, because the growth of the CCWs did not decrease when they were reared on an artificial diet together with cut goldenrod volatiles, it was concluded that the volatiles did not directly affect the performance of the CCWs. However, the growth of the CCWs was significantly decreased on soybean plants that had been exposed to cut goldenrod volatiles, indicating that undamaged soybean plants had better defenses against the larvae as a response to the previous volatile exposure. We also showed that at least α-pinene, β-myrcene, and limonene were involved in the defense induction. Thus, the results of the field experiments can, in part, be explained by this volatile compound-induced defense response. Similar no-choice feeding bioassays in plant–plant communication studies have been recently reported in the tomato (*Solanum lycopersicum*)–common cutworm (*S. litura*) and the willow (*Salix eriocarpa*)–willow leaf beetle (*Plagiodera versicolora*) systems^18^,^19^. In this study, in addition to the reduction of the relative growth ratio of CCWs, cut goldenrod volatiles might have acted as repellents of herbivores and attractants of natural enemies^1^,^2^. This possibility will be studied in the future.

Because daidzein, glycitein, and genistein are characteristic isoflavonoids of bean plants, we focused on these three isoflavones. Daidzein and glycitein restrict the growth of microbial pathogens^20^,^21^. Malonyl isoflavones are considered as storage forms in vacuoles, serving as a pool of precursors or inactive forms of phytoalexins^22^. We found that isoflavones, their glycosides and malonyl glycosides increased significantly in the seeds of exposed soybean plants. These compounds accumulated in soybean seeds through the phenylpropanoid pathway^17^,^23^. This was the first study of parental care in plant–plant communications where the maternal communication affected the accumulation of secondary compounds in the next generation (seeds). Detailed gene expression studies of the phenylpropanoid pathway, and work examining other secondary compounds produced by the phenylpropanoid pathway in seeds are beyond the scope of this study.

Weeding is an important and frequent component of agricultural and urban land use. In addition to the original purpose of weeding in agro-ecosystems, weeding resulted in the induction of defensive responses in soybean plants and activated the phenylpropanoid pathway in their seeds in a plant–plant communication context. The mechanisms involved in these responses will be studied in the future. Additionally, future research will evaluate the utility of these volatiles in pest management programs.

## Methods

### Field trial experimental designs

Field trials (2012 and 2013) were carried out at the Hyogo Prefectural Technology Center for Agriculture, Forestry and Fisheries. Soybean plants (*G. max* cv. Hyokei Kuro-3) were planted in a 40 m × 90 m field. The field was divided into seven plots (2 control, 2 treated, and 3 buffer), each with 180 plants (Figure S2). Plots were not treated with insecticides. Field trials (2015) were carried out at the Kyoto Prefectural Agriculture, Forestry and Fisheries Technology Center. Soybean plants were planted in a 50 m × 20 m field. The field was divided into four plots (2 control and 2 treated), each with 65 plants (Figure S2). Instead of establishing buffer plots, control and treated plots were divided by galvanized iron sheets (1 m × 15 m) to minimize the diffusion of volatiles. Plots were not treated with insecticides. Before the experiments, both fields were cultivated uniformly to exclude possible plot effects.

Soybean plants were grown on ridges. We earthed up soybean plants once during the experiments, and weeds were removed by this treatment. This was an agronomic system that requires no weeding. Thus, the weed conditions in the plots were identical. A chemical fertilizer was added to the field at the time of planting and then 2 weeks later because this is the agronomic practice for black soybeans.

When plants were 20–30 cm high, nine mesh bags (35 × 45 cm) containing cut goldenrod (~20-cm pieces, 500 g) were placed in each treated plot, and replaced twice during a 2–3-week period (Figure S1, Table S1). After exposure, plants were grown without insecticides or weeding. Near September (Table S1), the leaves on the 15 soybean plants in each of the plots (two treated and two control) were sampled to evaluate defoliation using the following levels: Level l (0–10%), Level 2 (10–25%) and Level 3 (>25%). In 2015, we observed whitened leaves damaged by CCWs, which was distinguishable from the damage caused by other herbivores by the naked eye.

In November, we randomly chose 15 soybean plants from each plot. We recorded the number of undamaged seeds, seeds with brown spots (made by stinkbugs), and seeds with damaged edges (made by lepidopteran larvae).

### Laboratory bioassay

To determine whether exposure to volatiles from goldenrod induced defense responses, two sets of 30 soybean seedlings (~20-cm high) were placed in acrylic cages (60 × 60 × 60 cm). In one cage there was a mesh bag containing cut goldenrod (300 g) hung from the ceiling, while in the other the bag was empty. After 4 days, we moved the plants into a climate-controlled room [25 °C ± 3 °C, under a 16 h L (100 μmol photons m^−2^ s^−1^):8 h D photoperiod) without goldenrod volatiles for 2 days. Then, each plant was infested with a second instar *S. litura* larva of known weight. Seven days later we compared the weight gain of larvae fed on exposed and control plants. We conducted similar experiments using a synthetic volatile blend as an odor source. The synthetic compounds in pure form (α-pinene, β-myrcene, and limonene) (Wako Chemicals Co. Ltd., Osaka, Japan) were dissolved in triethyl citrate (TEC). Using a gas chromatography analysis, the ratios of the compounds in the synthetic odor mixtures were tuned to those from an artificially damaged goldenrod plant. The synthetic mixture of pure α-pinene, β-myrcene, and limonene was achieved with a ratio of 20:1:1, respectively.

A dilution of 0.88% (w/w: α-pinene; 0.8%, β-myrcene: 0.04%, and limonene; 0.04%) of the synthetic mixture of the three compounds at the above ratio in TEC was prepared. We absorbed 1.7 g of this mixture in TEC into a piece of filter paper (22 mm × 35 mm, 2.8 mm thickness). To further facilitate a low volatilization rate, the piece of paper was covered with polyethylene film (30 × 40 mm, 100 μm thickness) (hereafter the dispenser bag). Five dispenser bags were connected to form one string. Two strings were used as an odor source. The concentrations of volatiles from the two strings were adjusted to be those normally emitted from cut plants. As control experiments, we used two strings into which only TEC was impregnated.

To test whether exposure to goldenrod volatiles affected the performances of CCWs we conducted a second series of experiments that involved rearing insects on an artificial diet (Insecta LF, Nosan Corporation, Yokohama, Japan) using the same protocol as above. However, in this case, individual second stadium larvae of a known mass were reared in separate petri dishes with mesh screens on top in the presence or absence of the volatile source for 7 days before they were reweighed.

### Volatile analysis

We passed air (100 mL/min airflow) through 100 g samples of cut goldenrod in polyethylene terephthalate bags (180 mm × 250 mm), and collected the headspace volatiles on porous Tenax TA polymer beads (100 mg, mesh 20/35, GL Sciences Inc., Tokyo, Japan) in a glass tube (3.0 mm internal diameter, 160 mm length) for 2 h and 0, 4, and 6 days after cutting (N = 4, at each time point). The collected volatiles were analyzed using a gas chromatograph–mass spectrometer (MS) with an HP-5MS capillary column (Agilent Technologies Inc., Santa Clara, CA, USA) equipped with a thermal desorption cold trap injector (TCT-CP4010, Chrompack, Middelburg, The Netherlands). The oven temperature of the gas chromatographer was programmed to rise from 40 °C (5-min hold) to 280 °C at 15 °C/min. The compounds were identified by comparing retention times and mass spectra with those in our own database or with those of synthetic compounds, except for thujene and (*Z*)-2-penten-1-yl acetate, which were not commercially available.

### Statistical analyses

To analyze the effects of the treatment, year and their interaction on the proportion of the leaf damage level, we used a GLMM with a binomial distribution and logit-link using the function glmer in package lme4 version 1.1.7^24^ in R version 3.1.1^25^. Although we maintained identical plot conditions before and during the experiments (see field trial experimental designs), individual plants would be exposed to different biotic/abiotic conditions. Since we sampled data from multiple leaves per plant, the plot and the individual plant were random effects in the models. To analyze the effects of the treatment, year and their interaction on the number of seeds, we used a GLMM with a normal distribution and identity-link with the function lmer in the package lme4. The plot was a random effect in the models. Significant values from the GLMMs were calculated from type II Wald chi-square tests using the function ‘Anova’ in the package car version 2.0.21^26^.

To analyze the effects of the treatment on the proportion of plants damaged by CCWs, we used a GLMM with a binomial distribution and logit-link with the function glmer in the package lme4. The plot was a random effect in the models. The significance of each model was evaluated using the likelihood ratio test, comparing models with or without the effects of the treatment.

To compare the weight gains of CCW larvae reared on treated and control plants, a one-way randomized-block ANOVA was used. The one-way randomized-block ANOVA was conducted using the JMP software package (version 11.2.1; SAS Institute, Cary, NC, USA). To determine whether there were differences in the amounts of each volatile compound emitted from cut goldenrods at 0, 4, 7 days after cutting, we used Welch’s one-way ANOVA in the JMP. Volatile compounds that were not detected in samples from any day were excluded from the analyses. To analyze the effects of the treatment on the amounts of isoflavones and the effects of the treatment, malonylation (glycoside or malonyl glycoside) and their interaction on the amounts of glycosylated isoflavones and malonyl glycosylated isoflavones, we used a GLM with a normal distribution and identity-link in JMP.

All data on seed numbers, as well as volatile compound and isoflavone levels, was Box-Cox transformed using JMP before statistical analyses. The amounts of α-pinene, sabinene, β-pinene and γ-terpinene showed normal distributions, and the fitting of the normal distribution was reduced when they were Box-Cox transformed. Thus, the amounts of these compounds were not Box-Cox transformed. All of the data on seed numbers and volatile compound levels were positively adjust by 0.5 before being Box-Cox transformed to avoid having values of 0.

### Liquid chromatography–photodiode array (PDA)/MS/MS analysis of isoflavones in soybean seeds

Soybeans were milled using an ultracentrifugal mill (Retsch ZM 1000; HAAN, Germany) with a 0.5 type mesh at 15,000 rpm. Isoflavones were extracted in a 5-fold volume of 80% (v/w) methanol at room temperature for 1 h. The crude extracts from soybean seeds were used directly in a UFLC system (Prominence UFLC system, Shimadzu, Kyoto, Japan) equipped with a PDA detector on a C-30 reverse-phase column (Develosil C30-UG-3, 2.0 mm i.d. × 150 mm; Nomura Chemical, Seto, Okayama, Japan) at 40 °C. Extracts from soybean seeds were diluted 100 times with 80% methanol prior to use for MS analysis in the UFLC system with PDA and a MS/MS (LTQ Orbitrap XL, Thermo Fisher Scientific, Waltham, MA, USA). The injection volume was 5 μL for each extract.

Solvent A was 0.1% formic acid solution, and solvent B was acetonitrile containing 0.1% (v/v) formic acid. A linear gradient elution of acetonitrile concentrations of 10% to 65% containing a constant 0.1% formic acid was performed at a flow rate of 0.15 mL/min: solvent B was initiated at 10% (v/v), increased to 65% (v/v) over 55 min, and then increased to 100% (v/v) for 5 min. The eluent composition was returned to the initial state of 10% (v/v) solvent B for 15 min. The eluate from the column was monitored by a PDA detector at UV 205, 260, and 292 nm by a MS/MS in the positive ion mode of electrospray ionization. An automatic full-scan mode over a mass-to-charge ratio (m/z) range of 50–2,000, and the top three ion-trap modes were used to acquire MS and MS/MS data. The UV and MS spectra were recorded and analyzed with Xcalibur software version 2.1 (Thermo Fisher Scientific). Isoflavone components were identified by MS analysis and MS/MS fragment profiles. When the identified component was pure, as indicated by an MS total scan and UV peak, its content was calculated from a standard curve based on the peak area of the standard (purified genistein monitored at 260 nm and saponin Bb monitored at 205 nm). The detection limit was 1 pmol per injection (0.1 mmol/100 g soybean).

## Additional Information

**How to cite this article**: Shiojiri, K. *et al*. Weeding volatiles reduce leaf and seed damage to field-grown soybeans and increase seed isoflavones. *Sci. Rep.* **7**, 41508; doi: 10.1038/srep41508 (2017).

**Publisher's note:** Springer Nature remains neutral with regard to jurisdictional claims in published maps and institutional affiliations.

## Supplementary Material

Supplementary Figures
