# Supplementary material for: Weeding volatiles reduce leaf and seed damage to field-grown soybeans and increase seed isoflavones
Source: Sci Rep. 2017 Jan 30;7:41508. doi: 10.1038/srep41508 (PMC5278389; doi:10.1038/srep41508)
Supplement: Supplementary Figures [file srep41508-s1.doc]

**Weeding volatiles reduce leaf and seed damage to field-grown soybeans and increase seed isoflavones**

Kaori Shiojiri^1,2,a^, Rika Ozawa^1^, Ken-Ichi Yamashita^3,b^, Masayoshi Uefune^4^, Kenji Matsui^5^, Chigen Tsukamoto^6^, Susumu Tokumaru^7^ and Junji Takabayashi^1^*

^1^Center for Ecological Research, Kyoto University, 2-509-3 Hirano, Otsu, Shiga 520-2113, Japan

^2^The Hakubi Center for Advanced Research, Kyoto University, Yoshida, Sakyo-ku, Kyoto 606-8501, Japan

^3^Hyogo Prefectural Technology Center for Agriculture, Forestry and Fisheries, Agricultural Technology Institute, Kasai 679-0198, Japan

^4^Department Agrobiological Resources, Faculty of Agriculture, Meijo University, Nagoya, Aichi 468-8502, Japan

^5^Graduate School of Sciences and Technology for Innovation (Faculty of Agriculture), Yamaguchi University, 1677-1 Yoshida, Yamaguchi 753-8515, Japan

^6^Graduate School of Agriculture, Iwate University, Ueda, Morioka 020-8550, Japan

^7^ Kyoto Prefectural Agriculture, Forestry and Fisheries Technology Center, Kameoka, Kyoto 621-0806, Japan

^a^Present address: Department of Agriculture, Ryukoku University, 1-5 Ooe, Otsu, Shiga 520-2194, Japan

^b^Present address: Bayer Crop Science, 2-4-9 Umeda, Kitaku, Osaka 530-0001, Japan


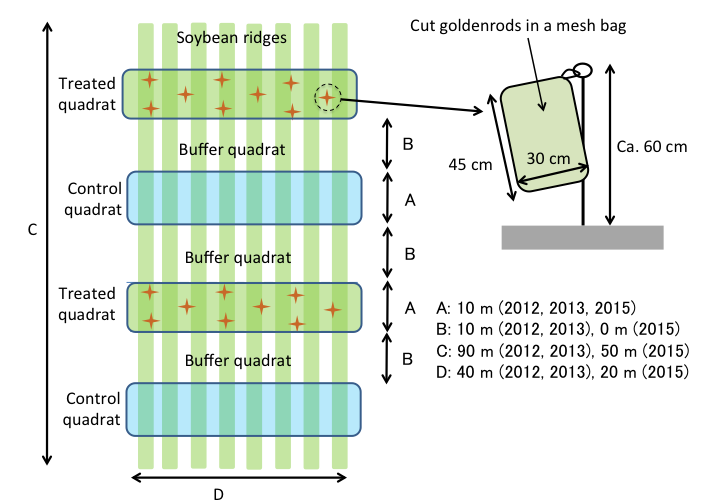


Figure S1. Design of field experiments

**
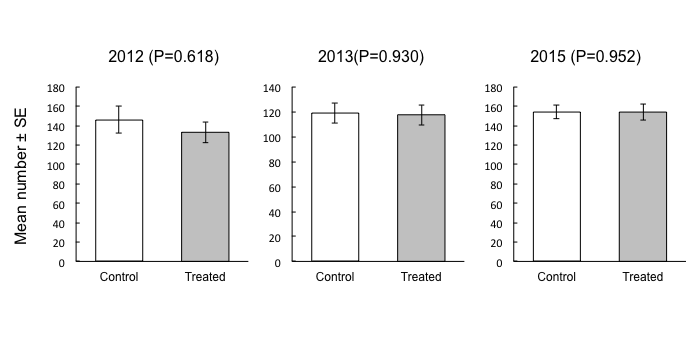
**

Figure S2. Total number of seeds harvested. Treated: soybean plants exposed to volatiles from cut golden rods. Control: unexposed. GLMMs were applied to estimate the effects of the treatment on the total number of seeds in each year.

Table S1. Experimental soybean field-related dates

|  | Duration of the exposure of cut goldenrod volatiles | Date of observation of damaged leaves | Harvest date |
| --- | --- | --- | --- |
| 2012 | July 24 to August 13 | September 1 | November 27 |
| 2013 | June 27 to July 19 | September 12 | December 9 |
| 2015 | June 24 to July 8 | September 10 | November 27 |
